# Periplasmic α-carbonic anhydrase plays an essential role in *Ralstonia eutropha *CO_2 _metabolism

**DOI:** 10.1186/1753-6561-8-S4-P183

**Published:** 2014-10-01

**Authors:** Claudia Gai, Jingnan Lu, Christopher Brigham, Amanda Bernardi, Anthony Sinskey

**Affiliations:** 1 Department of Biology, Massachusetts Institute of Technology, 77 Massachusetts Avenue, Cambridge, MA 02139, USA; 2 Department of Chemistry, Massachusetts Institute of Technology, 77 Massachusetts Avenue, Cambridge, MA 02139, USA; 3 Department of Bioengineering, University of Massachusetts Dartmouth, 285 Old Westport Road, North Dartmouth, MA 02747, USA; 4 Department of Biology, Massachusetts Institute of Technology, Bldg. 68-370, 77 Massachusetts Avenue, Cambridge, MA 02139, USA; 5 Department of Biology, Division of Health Sciences and Technology, Engineering Systems Division, Massachusetts Institute of Technology, 77 Massachusetts Avenue, Cambridge, MA 02139, USA

## Background

Carbonic anhydrase (CA) enzymes catalyze the interconversion of CO_2 _and bicarbonate. These enzymes play important roles in cellular metabolism such as CO_2 _transport, ion transport, and internal pH regulation. Understanding the roles of CAs in the chemolithotropic betaproteobacteria *Ralstonia eutropha *is important for the development of fermentation processes based on the bacterium's capacity for carbon fixation using the Calvin-Benson-Bassham cycle. Of the five classes of CA, the alpha-CA is the best-characterized thus far. The gene encoding a periplasmic alpha-CA (*caa*, H16 B2403) has been identified in the *R. eutropha *H16 genome, along with three others CA from different classes. In this study, we evaluated the importance of Caa in the metabolism of *R. eutropha *by examination of CA activity and growth in *caa *gene deletion, complementation, and overexpression strains. Localization of Caa in the cell was accessed by fluorescent microscopy.

## Methods

*Ralstonia eutropha *(H16) strains were propagated in TSB or mineral medium [1].

Standard protocols were employed for plasmid and strain construction [1,2]. Purification of tagged-proteins was performed following manufacturer's instructions.

The CA activity assays were based on protocols from Sundaram et al. [3] and Fasseas et al. [4] with some modifications.

Cells were observed under 100X magnification using a Nikon Labophot-2 microscope with phase-contrast and fluorescence attachments.

## Results and conclusions

Purified Caa was found capable of performing the interconversion of CO_2 _and HCO_3_^- ^with equilibrium lying towards HCO_3_^- ^formation. The activities detected were 83.6 + 14.4 EU/mg protein and 422.2 + 97.1 EU/mg protein using bicarbonate and CO_2 _as substrates respectively.

Deletion of *caa *(strain Re2428 (H16 Δ*caa*)) resulted in poor growth in all conditions tested, even with addition of CO_2 _to culture.

In an attempt to recover *R. eutropha *growth and to determine the effect of Caa localization on cell growth the annotated gene with (caa) and without (caaB) the nucleotide sequence encoding the 23-aa N-terminal predicted signal-peptide were amplified, cloned separately into expression vectors and reintroduced *in trans *into Re2428.

Overall, reintroduction of *caa *or *caaB *in Re2428 was able to recover only partial growth when compared to the wild type. However, the strain Re2428/pCaa presented better growth when compared to Re2428/CaaB in mineral media. This could be an effect of the different localization of Caa in the cell. Caa deletion and mislocalization adversely affected cell growth by causing damage to metabolism. The periplasmic localization of Caa was confirmed by microscopy using the red fluorescent protein (RFP)-tagged enzyme.

We demonstrated the importance of Caa for the transport of CO_2 _and the supply of bicarbonate to *R. eutropha *cells, as its correct localization and balanced expression in the cell are essential for *R. eutropha *during growth. The localization of Caa in the periplasm of the cell is crucial for the conversion of CO_2 _to bicarbonate since Caa is probably responsible for rescuing the CO_2 _which diffuses into the cell and converting it to bicarbonate then transporting it across the cell membrane back to central metabolism.
